# Factors affecting lymphedema after neoadjuvant chemotherapy and axillary dissection in female breast cancer patients: a retrospective cohort study based on the Chinese population

**DOI:** 10.3389/fonc.2024.1436748

**Published:** 2024-10-29

**Authors:** Jianqin Fu, Ruiliang Chen, Lijuan He, Liqun Bao, Zhaodi Lin, Weijing Jiang, Jie Zhang, Chuan Wang, Yanjuan Lin

**Affiliations:** ^1^ Department of Breast Surgery, Fujian Medical University Union Hospital, Fuzhou, Fujian, China; ^2^ Department of Breast Surgical Nursing, Fujian Medical University Union Hospital, Fuzhou, Fujian, China; ^3^ Department of General Surgery, Fujian Medical University Union Hospital, Fuzhou, Fujian, China; ^4^ Breast Cancer Institute, Fujian Medical University, Fuzhou, Fujian, China; ^5^ Department of Nursing, Fujian Medical University Union Hospital, Fuzhou, Fujian, China

**Keywords:** breast cancer, lymphedema, risk factors, ALND, neoadjuvant chemotherapy

## Abstract

**Purpose:**

Breast cancer-related lymphedema (BCRL) is a common complication among breast cancer survivors. Most BCRL studies have focused on patients receiving adjuvant chemotherapy, with relatively little attention paid to BCRL in patients undergoing neoadjuvant chemotherapy (NAC). This study aimed to investigate the risk factors associated with BCRL in Chinese women undergoing NAC and axillary lymph node dissection (ALND).

**Methods:**

At our institution, this cohort study collected data from 336 women with breast cancer and documented axillary nodal metastasis at diagnosis, who received NAC and ALND surgery between 2015 and 2020. BCRL was assessed through both objective limb circumference measurements and subjective self-reported symptoms. Multivariate logistic regression was employed to identify risk factors for BCRL, considering clinical, demographic, and lifestyle-related characteristics.

**Results:**

The cumulative incidence of BCRL within 2.5 years was 43.75%. Factors independently associated with BCRL included radiotherapy (versus no radiotherapy; hazard ratio (HR) = 1.611; P = 0.020), NAC duration of 105 days or shorter (versus 105-143 days; HR = 0.471; P = 0.020), removal of more than 15 lymph nodes (versus 15 or fewer lymph nodes; HR = 1.593; P = 0.036), drainage duration of 20-29 days (versus 10-19 days; HR = 1.568; P = 0.028), and sleeping biased toward the affected arm (versus sleeping biased toward the healthy arm; HR = 2.033; P = 0.019).

**Conclusion:**

This study identified several risk factors for BCRL in breast cancer patients following NAC and ALND. Patients presenting with one or more of these factors should be monitored closely for early detection and intervention. Further research is warranted to explore the impact of drainage duration and sleep position on the development of BCRL.

## Introduction

Breast cancer survival rates have increased significantly in recent decades due to improvements in screening and advances in multidisciplinary treatment (1). Therefore, maintaining the quality of life and controlling treatment-related complications in long-term survivors has become an important goal (2–5). Breast cancer-related lymphedema (BCRL) is a common comorbidity of the upper extremity secondary to breast cancer treatment that occurs in approximately 22% of survivors (6). Additionally, a report indicates that BCRL might manifest anytime between the initial treatment and up to 20 years post-surgery, with the majority of cases occurring within the first 3 years (7). BCRL occurs when protein-rich fluid accumulates in the soft tissues caused by an interruption of lymphatic flow, which negatively affects the patient’s quality of life, both physically and psychosocially (8, 9).

Current data suggest that the development of BCRL is multifactorial and influenced by three categories of factors: disease and treatment-related factors (such as tumor size, axillary lymph node dissection [ALND] surgery, chemotherapy, and radiotherapy), lifestyle factors (such as physical activity, body mass index [BMI], and preventive behaviors, and demographic factors (such as monthly income, marital status, and ethnicity) (9–15). In addition to established risk factors, our study identified two rarely reported independent factors: postoperative sleeping position and drainage duration (16). To the best of our knowledge, no previous studies have specifically explored the link between sleeping position and BCRL.

Neoadjuvant chemotherapy (NAC) is increasingly used in treating breast cancer because of its ability to downstage the primary tumor in the breast and the metastatic axillary lymph node (17). However, NAC has recently been recognized as an independent risk factor for BCRL (18). On the other hand, the advent of sentinel lymph node biopsy (SLNB) has resulted in lower lymphedema rates by avoiding unnecessary ALND (19). But ALND remains the standard of axillary surgery in patients with clinically positive lymph nodes or metastatic sentinel nodes (20). Therefore, women who have undergone NAC and ALND surgery are at significant risk of developing lymphedema. However, few studies have simultaneously investigated the demographic, disease and treatment-related, and lifestyle factors that predict the development of BCRL in this subset of patients.

Breast cancer survivors who have been provided with BCRL information have significantly reduced symptoms and increased knowledge of BCRL (21). Therefore, this study was carried out to identify potential risk factors for the occurrence of BCRL in breast cancer patients who received NAC and ALND, with the aim of optimizing lymphedema surveillance and improving patient education on BCRL.

## Methods

### Eligibility criteria

This cohort study enrolled 354 newly diagnosed breast cancer patients who had undergone primary breast cancer surgery from June 2015 to June 2020 at Fujian Medical University’s Union Hospital. The inclusion criteria were as follows: (1) 18 years of age or older, (2) AJCC clinical T0-4N1-2M0 breast cancer patients who underwent fine-needle aspiration or core needle biopsy of an axillary node with documented nodal metastasis at diagnosis, prior to neoadjuvant chemotherapy, (3) data available at baseline and at least one post-operative follow-up time point, (4) received NAC and subsequent ALND surgery. Exclusion criteria were as follows: (1) bilateral breast cancer, (2) existing arm edema before surgery, (3) presence of severe cardiac or renal disease, (4) local or systemic recurrence of breast cancer. Finally, all 336 patients who received NAC and subsequent breast surgery and ALND were successfully included in the cohort. The hospital ethics committee approved the protocol, and informed consent was obtained from all study patients.

### Measurement and assessment of lymphedema

Breast cancer-related lymphedema is diagnosed by objective measurement of limb circumference and subjective assessment (self-reported symptom). Measurements were obtained at the following time points: before surgery (after completion of neoadjuvant chemotherapy) (baseline), and 1, 3, 6, 12, 18, 24, and 30 months after surgery. The professionally trained nurse measured the limb circumference of both arms with flexible tape at four anatomical locations: (1) metacarpal, (2) wrist, (3) 10 cm below the lateral condyle, and (4) 10 cm above the lateral condyle, with the patient in a standing position with the elbow extended and the forearm in flexion (22). Additionally, patients were asked at each follow-up visit whether they were currently experiencing swelling, heaviness, numbness, tightness, or pain in the affected arm.

The patient was diagnosed with BCRL if the circumference of the affected arm (arm on the side where the axillary dissection was performed) was greater than 2 cm at one or more anatomical locations compared to baseline and the contralateral arm. The formula for calculating the increase in arm circumference was as follows: (ipsilateral time point value - ipsilateral baseline value) - (contralateral time point value - contralateral baseline value) (23). Additionally, patients who reported at least one of four self-reported arm symptoms (swelling, heaviness, tightness, or numbness), but with less than a 2 cm interlimb difference, were also considered to have lymphedema (24). The time to develop BCRL was calculated from the date of the defined breast surgery to the date of BCRL diagnosis (25).

### Statistical analysis

The aim of this analysis was to assess the risk factors associated with lymphedema. Categorical variables were presented as the number of patients (%) and differences between the two groups were assessed using the χ2 and Fisher’s exact tests. The cumulative risk of various factors was determined using univariate logistic regression analysis. Factors associated with the development of lymphedema were analyzed using univariate logistic regression, without considering the interference of other factors. Variables with p-values < 0.1 in univariate analysis were included in multivariate logistic regression. After adjusting for other factors, multivariate logistic regression was employed to identify statistically significant risk factors correlated with lymphedema development. A statistically significant difference was defined as a p-value less than 0.05 in multivariate analysis. Point estimates (e.g., percentage of patients, hazard ratio [HR], and 95% confidence interval [CI] were used to summarize variables and correlations. All statistical analyses were performed using SPSS software version 25.

## Result

This study involved 336 patients with unilateral breast cancer, of whom 147 (43.75%) had BCRL. The comparison of demographics, clinical, and lifestyle characteristics between the BCRL and non-BCRL cohorts is presented in **Table 1**. Patients with BCRL exhibited higher BMI, more positive lymph nodes, and a greater number of lymph nodes removed. They were also more likely to undergo radiotherapy and longer NAC treatment compared to those without BCRL. Additionally, significant differences (P < 0.05) were observed in the following variables: home care, TNM staging, duration of drainage, postoperative sleeping position and postoperative drainage volume.

**Table 1:** Baseline Characteristics by Status.ª.

Characteristic	Non-lymphedema group (n=187)	Lymphedema group (n=149)	P Value
Age, years			
<50 ≥50	89 (58.6%) 98 (53.3%)	63 (42.4%) 86 (46.7%)	0.331
BMI category			
<24	121 (62.7%)	72 (37.3%)	0.003
≥24	66 (46.2%)	77 (53.8%)	
Education level			
University	42 (66.7%)	21 (33.3%)	0.131
Secondary school	78 (54.5%)	65 (45.5%)	
Elementary school and below	67 (51.5%)	63 (48.5%)	
Pressure sleeve			
Yes	45 (58.4%)	32 (41.6%)	0.575
No	142 (54.8%)	117 (45.2%)	
Rehab exercise			
Yes	80 (58.8%)	56 (41.2%)	0.335
No	107 (53.5%)	93 (46.5%)	
Monthly income, RMB			
<3000	109 (54.8%)	90 (47.2%)	0.695
≥3000	78 (56.9%)	59 (43.1%)	
Home care			
Spouse	104 (54.2%)	88 (45.8%)	0.027
Children	64 (62.1%)	39 (37.9%)	
Others	18 (56.3%)	14 (43.7%)	
No	1 (11.1%)	8 (88.9%)	
Surgery side			
Left	87 (55.8%)	69 (44.2%)	0.969
Right	100 (55.6%)	80 (44.4%)	
Surgery on dominant side			
Yes	101 (54.9%)	83 (45.1%)	0.825
No	86 (56.6%)	66 (43.4%)	
Surgery type			
Mastectomy	172 (54.8%)	142 (45.2%)	0.221
BCS	15 (68.2%)	7 (31.8%)	
Tumor location			
upper-inner quadrant	31 (55.3%)	25 (44.7%)	0.517
lower-inner quadrant	10 (58.8%)	7 (41.2%)	
upper-outer quadrant	103 (52.6%)	93 (47.4%)	
lower-outer quadrant	27 (67.5%)	13 (32.5%)	
central Quadrant	16 (59.3%)	11 (40.7%)	
TNM stage			
YPCR	4 (80.0%)	1 (20.0%)	0.001
I	7 (87.5%)	1 (12.5%)	
II	128 (61.5%)	80 (38.5%)	
III	47 (41.6%)	66 (58.4%)	
IV	1 (50.0%)	1 (50.0%)	
Total nodes removed			
≤15	70 (72.2%)	27 (27.8%)	<0.001
>15	117 (49.0%)	122 (51.0%)	
Total positive nodes			
0	4 (66.7%)	2 (33.3%)	0.006
1-3	133 (61.6%)	83 (38.4%)	
≥4	50 (43.9%)	64 (56.1%)	
Radiotherapy			
Yes	99 (47.8%)	108 (52.2%)	<0.001
No	88 (68.2%)	41 (31.8%)	
Endocrine therapy regimen			
No	72 (50.0%)	72 (50.0%)	0.101
SERM	17 (70.8%)	7 (29.2%)	
AI	98 (58.3%)	70 (41.7%)	
Neoadjuvant chemotherapy regimen			
Taxane-containing regimen	62 (59.6%)	42 (40.4%)	0.068
Anthracycline-containing regimen	9 (52.9%)	8 (47.1%)	
Anthracycline- and taxane-containing regimen	98 (51.0%)	94 (49.0%)	
Other	18 (78.3%)	5 (21.7%)	
Duration of NAC, days			
<105	51 (78.5%)	14 (21.5%)	<0.001
105-143	50 (48.1%)	54 (51.9%)	
>143	86 (51.5%)	81 (48.5%)	
Duration of Drainage, days			
<10	6 (42.9%)	8 (57.1%)	<0.001
10-19	129 (62.0%)	79 (48.0%)	
20-29	31 (36.0%)	55 (64.0%)	
>29	21 (75.0%)	7 (25.0%)	
Postoperative sleeping position			
Biased to the healthy side	49 (61.3%)	31 (38.7%)	<0.001
Biased to the surgery side	7 (18.0%)	32 (72.0%)	
Lied flat	42 (56.8%)	32 (43.2%)	
Alternated	89 (62.2%)	54 (37.8%)	
Postoperative drainage volume,ml			
<200	121 (60.5%)	79 (39.5%)	0.030
≥200	66 (48.5%)	70 (51.5%)	

BMI, body mass index (calculated as weight in kilograms divided by height in meters squared); BCS, breast conservation surgery; TNM, Tumor Node Metastasis; YPCR, pathological complete response; NAC, neoadjuvant chemotherapy. SERM, selective estrogen receptor modulator; AI, aromatase inhibitor.

ªData are presented as number(percentage) of patients.

**Figure 1:**
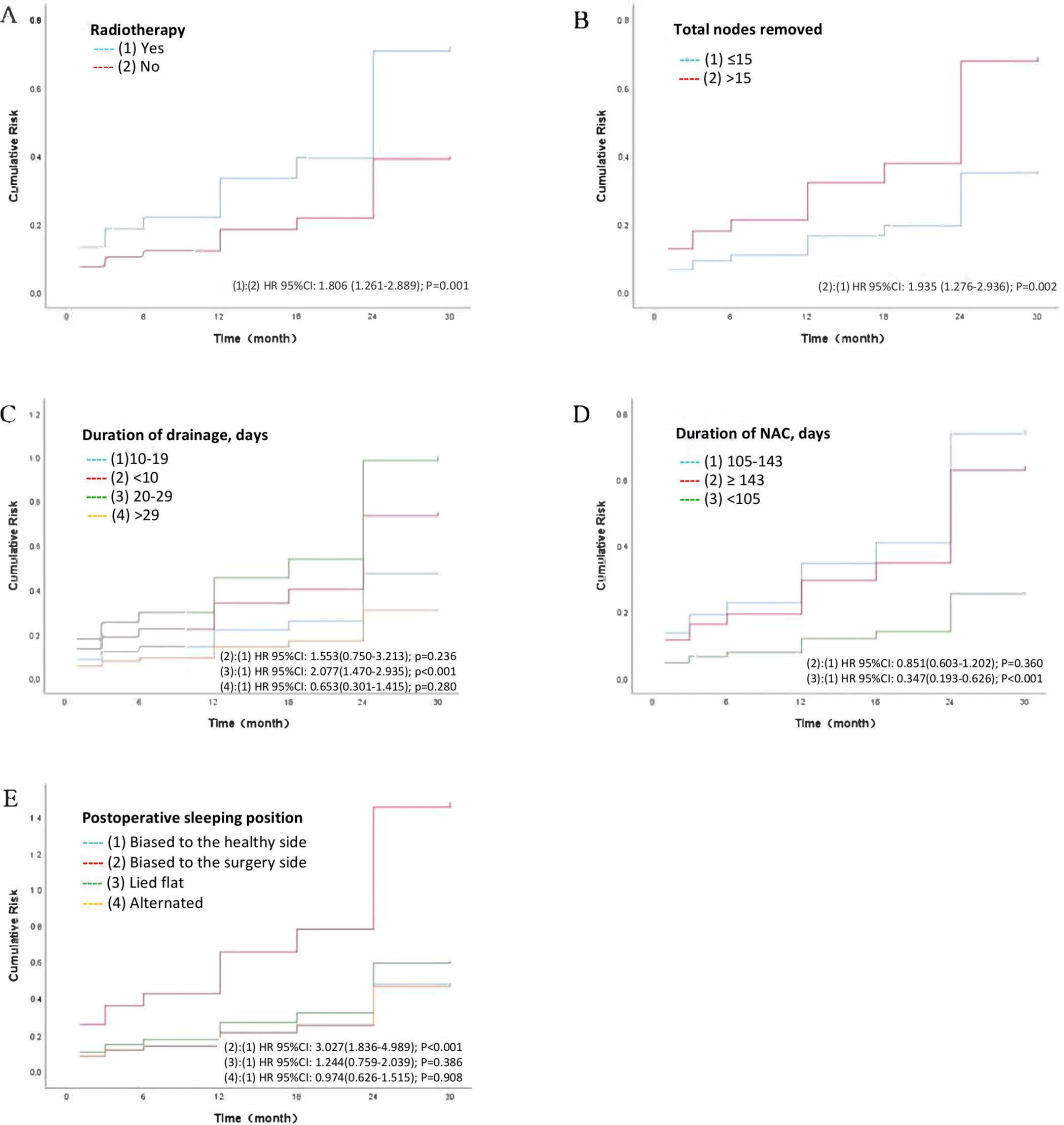
Lymphedema cumulative risk based on the independent risk factors. **(A)** The cumulative risk of lymphedema according to radiotherapy. **(B)** The cumulative risk of lymphedema according to the total number of nodes removed. **(C)** The cumulative risk of lymphedema according to the duration of drainage. **(D)** The cumulative risk of lymphedema according to the duration of NAC. **(E)** The cumulative risk of lymphedema according to the postoperative sleeping position. NAC, neoadjuvant chemotherapy; HR, hazard ratio; CI, confidence interval.

In the univariate analysis, ten factors were correlated with the development of BCRL and were included in the multivariate analysis (**Table 2**). The multivariate analysis identified five factors as independent predictors of BCRL (P < 0.05): (1) The BCRL rates significantly increased in relation to the total lymph nodes removed >15 (HR = 1.593; P = 0.036). (2) Radiotherapy was associated with a higher rate of BCRL (HR = 1.611; P = 0.020). (3) The BCRL rates were lower among patients who received NAC for 105 days or shorter (HR = 0.471; P = 0.020). (4) Women with drainage for 20-29 days were found to be at a significantly increased risk of developing BCRL compared to those with drainage for 10-19 days (HR = 1.568; P = 0.028). (5) The HR of BCRL for the postoperative sleeping position (biased to the surgery side versus biased to the healthy side) was 2.033 with P = 0.019. However, after adjusting for other variables, BMI, postoperative drainage volume, home care, total positive nodes, and endocrine therapy were not statistically significant in the multivariate analysis. The 2.5-year cumulative risk of BCRL according to the five independent risk factors that were found to be significantly different in both univariate and multivariate analyses (radiotherapy, total lymph nodes removed, duration of NAC, duration of drainage, and postoperative sleeping position) is presented in **Figure 1**.

**Table 2:** Univariate and multivariate analysis of risk factors associated with lymphedema.ª.

Variable	HR (95%CI)	P Value
Univariate Analysis		
Age, years		
<50	1 [Reference]	
≥50	1.195 (0.863-1.654)	0.283
BMI category		
<24	1 [Reference]	
≥24	1.502 (1.089-2.072)	0.013
Home care		
No	1 [Reference]	
Spouse	0.380 (0.183-0.787)	0.009
Children	0.301 (0.140-0.647)	0.002
Others	0.359 (0.150-0.858)	0.021
Total lymph nodes removed		
≤15	1 [Reference]	
>15	1.935 (1.275-2.936)	0.002
Total positive lymph nodes		
0	0.516 (0.126-2.106)	0.356
1-3	0.604 (0.436-0.838)	0.002
≥4	1 [Reference]	
Radiotherapy received		
No	1 [Reference]	
Yes	1.806 (1.261-2.589)	0.001
TNM stage		
YPCR	1 [Reference]	
I	0.693 (0.043-11.077)	0.795
II	2.277 (0.317-16.368)	0.413
III	3.883 (0.539-27.984)	0.178
IV	3.368 (0.211-53.872)	0.391
Duration of NAC, days		
<105	0.347 (0.193-0.625)	<0.001
105-143	1 [Reference]	
≥144	0.851 (0.603-1.202)	0.360
Duration of Drainage, days		
<10	1.553 (0.750-3.213)	0.236
10-19	1 [Reference]	
20-29	2.077 (1.470-2.935)	<0.001
>29	0.653 (0.301-1.415)	0.280
Postoperative sleeping position		
Biased to the healthy side	1 [Reference]	
Biased to the surgery side	3.027 (1.836-4.989)	<0.001
Lied flat	1.244 (0.759-2.039)	0.386
Alternated	0.974 (0.626-1.515)	0.908
Postoperative drainage volume, ml		
<200	1 [Reference]	
≥200	1.420 (1.029-1.960)	0.033
Endocrine therapy		
No	1 [Reference]	
SERM	0.515 (0.237-1.119)	0.094
AI	0.790 (0.568-1.097)	0.159
Neoadjuvant chemotherapy regimen		
Taxane-containing regimen	1 [Reference]	
Anthracycline-containing regimen	1.189 (0.558-2.533)	0.653
Anthracycline- and taxane-containing regimen	1.155 (0.803-1.662)	0.437
Other	0.467 (0.185-1.179)	0.107
Multivariate Analysis		
Total lymph nodes removed		
≤15	1 [Reference]	
>15	1.593 (1.031-2.463)	0.036
Radiotherapy received		
No	1 [Reference]	
Yes	1.611 (1.079-2.406)	0.020
Duration of NAC, days		
<105	0.471 (0.249-0.890)	0.020
105-143	1 [Reference]	
≥144	0.724 (0.498-1.051)	0.090
Duration of Drainage, days		
<10	1.896 (0.896-4.014)	0.094
10-19	1 [Reference]	
20-29	1.568 (1.049-2.345)	0.028
>29	0.685 (0.310-1.514)	0.350
Postoperative sleeping position		
Biased to the healthy side	1[Reference]	
Biased to the surgery side	2.033(1.124-3.677)	0.019
Lied flat	1.171(0.705-1.946)	0.542
Alternated	1.044(0.729-1.495)	0.817

HR, hazard ratio; BMI, body mass index (calculated as weight in kilograms divided by height in meters squared); TNM, Tumor Node Metastasis; YPCR, pathological complete response; NAC, neoadjuvant chemotherapy; SERM, selective estrogen receptor modulator; AI, aromatase inhibitor.

## Discussion

The aim of this study was to explore potential risk factors for the development of BCRL in breast cancer patients who received NAC and ALND. Five independent risk factors were identified, including radiotherapy, duration of NAC, number of excised lymph nodes, duration of drainage, and postoperative sleeping positions. Our research encompasses a substantial dataset derived from a considerable patient cohort, coupled with a meticulously followed-up period. We believe that our findings could provide credible evidence prompting alterations in management strategies and enhancing patient care in this context.

The placement of a closed-suction drain in the mastectomy site and axilla after breast cancer surgery aims to decrease postoperative complications, particularly seroma formation (26–28). However, in our study, we found that patients with a longer duration of drainage were at a higher risk of BCRL. Similarly, Saadet et al. stated that the long duration of the axillary drain was a risk factor for BCRL (P = 0.045) (16). Two reasons may explain this phenomenon. Firstly, a longer duration of drainage reflects a higher degree of lymphatic vessel damage, supporting the notion that more extensive axillary surgery increases the incidence of BCRL (29). Secondly, patients with drainage tubes need to immobilize the affected limb to reduce drainage volume (30). However, carrying the drainage tube for a long time can lead to stiffness in the arm, causing the optimal time for postoperative limb rehabilitation exercises to be missed. A cross-sectional study of 775 patients showed that women who exercised their affected arm decreased the risk of developing BCRL through a potential mechanism called the “muscle pump” (15, 31, 32). At the same time, Our results showed that the >29 days drainage group had the lowest likelihood of lymphedema, which appears contradictory but is not statistically significant, likely due to the small sample size in this group.

A meta-analysis reported that the decision to remove drainage based on the amount of drainage would reduce the incidence of seroma, an independent risk factor for lymphedema, compared to short-term removal of drainage (33, 34). However, due to management regarding drain placement, the number of drains, and hospitalization varying widely between breast units (35), there are no widely applicable criteria for the removal of drainage. Therefore, future multicenter and larger cohort studies based on uniform criteria are required to better understand the relationship between drainage time and BCRL.

The relationship between postoperative sleeping position and BCRL has never been studied before. Our study found that sleeping biased towards the affected arm significantly increased the incidence of BCRL (HR = 2.033; p = 0.019). Prolonged compression of the affected limb impedes the return of lymphatic fluid, disrupting the morphology and function of the lymphatic system and ultimately leading to BCRL. Furthermore, prolonged compression of the limb leads to ischemia of the subcutaneous tissues, causing a reduction in subcutaneous fat and muscle atrophy, which further affects the functional recovery of the lymphatics. Patients often consciously avoid putting pressure on the affected arm in the early postoperative period. However, later in life, they may think they have recovered from breast cancer and may unconsciously sleep on the affected side. Additionally, postoperative sleeping position may be related to whether the surgery was performed on the dominant hand or not, but a chi-square test showed no statistical difference between the two (p = 0.252). This finding suggests that some breast cancer survivors overlook the negative impact of common lifestyle habits on BCRL, highlighting the importance of correcting postoperative sleep position.

The association between radiotherapy and BCRL is well documented in the literature (24, 25, 36, 37). In our study, patients who received radiotherapy were 1.8 times more likely to develop BCRL than those who did not. A retrospective study of 7,617 patients showed that patients with more extensive radiation fields were at greater risk of lymphedema; compared with no radiation or breast/chest wall radiation alone, regional lymph node irradiation (RNI) increased the risk of BCRL by 2-4 times (24). Additionally, the total number of lymph nodes removed is another well-known independent risk factor for BCRL (6, 9, 38, 39). Hwa Kyung Byun et al. reported that the 3-year cumulative BCRL rates were 3.0%, 10.0%, 20.2%, and 24.4% in patients with 0 to 5, 6 to 10, 11 to 15, and >15 lymph nodes removed, respectively (P < 0.001) (24). Interestingly, several reports suggest that the combination of ALND and radiotherapy has a synergistic effect on the development of BCRL (9, 25, 36). However, the relationship between radiotherapy regimens and the number of lymph nodes removed has rarely been studied, which may be helpful in developing individualized radiotherapy regimens for breast cancer patients receiving axillary dissection to reduce the incidence of BCRL.

In recent years, increasing attention has focused on studying the risk factors for BCRL in the NAC setting. Giacomo Montagna et al. found that NAC was an independent risk factor for BCRL (OR = 2.10; 95%CI = 1.16-3.95; P = 0.01) (40). Our study suggests a lower incidence of BCRL in patients with a shorter duration of NAC, which is in line with a study reporting that a longer NAC duration was correlated with increased BCRL incidence (18). In general, two possible factors contribute to this result. Firstly, the number of cycles of chemotherapy infusion in the ipsilateral arm was reported as an independent risk factor for developing BCRL by José Luiz B. Bevilacqua et al. (41). Secondly, regarding the specific toxicity of chemotherapy agents, many studies showed that taxane-based chemotherapy could result in BCRL by increasing extracellular fluid accumulation (42–44). Therefore, more attention should be paid to patients with a longer duration of NAC and those treated with taxane-based chemotherapy.

The strength of our study lies in the collection of bilateral arm measurements at multiple time points over a 30-month period, including preoperative measurements, which helps control for potential size differences between the dominant and non-dominant arms. However, the study has the following limitations: Firstly, in the absence of a gold standard, the diagnosis of BCRL in our study was based on a 2-cm difference in circumference and self-reported symptoms, which are inherently flawed and diagnostically imprecise, potentially leading to misdiagnosis. Secondly, we only looked at whether radiotherapy was given and did not analyze the effect of different radiotherapy regimens, which may limit the applicability of our results to more recently established treatments. However, many studies have shown that RNI is associated with a higher risk of developing BCRL than radiation to the breast or chest wall alone (24, 25). Finally, the overall rate of BCRL may be underestimated due to loss to follow-up, as some participants did not complete all scheduled assessments, leading to incomplete data. Future studies with more comprehensive follow-up strategies are necessary to mitigate the impact of loss to follow-up and provide a more accurate estimate of BCRL rates.

## Conclusion

In conclusion, our research showed that more than 40% of breast cancer patients who received NAC and ALND suffered from BCRL. We identified five independent risk factors associated with the development of BCRL: radiotherapy, duration of NAC, number of lymph nodes removed, duration of drainage, and postoperative sleeping position. Healthcare workers should focus on monitoring patients with one or more of these factors to enable early detection and intervention. Further research is needed to investigate the effects of drainage time and sleep position on the development of BCRL.

## Data Availability

The raw data supporting the conclusions of this article will be made available by the authors, without undue reservation.
